# The association between depressive symptoms from early to late adolescence and later use and harmful use of alcohol

**DOI:** 10.1007/s00787-014-0600-5

**Published:** 2014-08-18

**Authors:** Alexis C. Edwards, Carol Joinson, Danielle M. Dick, Kenneth S. Kendler, John Macleod, Marcus Munafò, Matthew Hickman, Glyn Lewis, Jon Heron

**Affiliations:** 1 Virginia Institute for Psychiatric and Behavioral Genetics, Virginia Commonwealth University, Richmond, VA USA; 2 School of Social and Community Medicine, University of Bristol, Oakfield House, Oakfield Grove, Clifton, Bristol, BS8 2BN UK; 3 School of Experimental Psychology, University of Bristol, Bristol, UK; 4 Mental Health Sciences Unit, University College London, London, UK

**Keywords:** ALSPAC, Adolescence, Depressive symptoms, Alcohol, Longitudinal

## Abstract

Depressive symptoms and alcohol misuse contribute substantially to the global health burden. These phenotypes often manifest, and frequently co-occur, during adolescence. However, few studies have examined whether both baseline levels of depressive symptoms and change in symptoms are associated with alcohol outcomes. In addition, inconsistent findings could be due to sex differences or the use of different alcohol outcomes. Using data from a prospective population-based cohort in the UK, we estimated trajectories of depressive symptoms from 12 years 10 months to 17 years 10 months, separately for male and female participants. We assessed whether baseline and change in depressive symptoms were associated with use and harmful use of alcohol at 18 years 8 months. Among females, increasing depressive symptoms were associated with increased alcohol use; whilst for males, there was little evidence of this. When examining harmful levels of alcohol use, baseline levels of depressive symptoms in males were weakly related to later harmful alcohol use but this association was attenuated substantially through adjustment for confounders. In contrast, both baseline symptoms and increase in symptoms were associated with later harmful alcohol use in females and these associations were not diminished by confounder adjustment. Elevated depressive symptoms during adolescence are positively associated with increases in both use and harmful use of alcohol at 18 years 8 months. These findings differ between the sexes. Further research is needed to examine the mechanisms underlying the link between depressive symptoms and harmful alcohol use to identify potentially modifiable factors for intervention.

**Electronic supplementary material:**

The online version of this article (doi:10.1007/s00787-014-0600-5) contains supplementary material, which is available to authorized users.

## Introduction

Alcohol misuse and depressive symptoms both contribute substantially to the global disease burden and adolescence is considered a high-risk period for the onset of these problems [1]. Furthermore, alcohol problems and symptoms of depression in adolescence can be precursors to problems during adulthood [2]. The relationship between depression and alcohol use, particularly during adolescence, is not consistent across studies [3], and has been less well characterized than the link between externalizing symptoms and alcohol problems [4]. Increased understanding of the aetiological basis for the association between depression and alcohol use phenotypes could provide further information on the benefits of interventions targeting alcohol use or depression during the critical adolescent developmental period.

An ‘internalizing’ subtype of alcohol dependency has been described previously [5, 6] although much of this earlier work has been conducted in adults. There is evidence, however, that a positive relationship between depressive symptoms and alcohol use/misuse becomes evident earlier in life, and extends to subclinical levels of problems. For example, using a method similar to growth curve modelling, Marmorstein [3] examined a population-based sample of adolescents and young adults, and found that subclinical depressive symptoms and alcohol problems had reciprocal effects on one another during this period, with some differences across the sexes. A study of younger adolescents, using an auto-regressive cross-lag model across four waves of data, suggested that depressive symptoms were positively associated with later alcohol use [7]. Many previous studies [8–11] of the association between depressive symptoms and alcohol use rely on cross-sectional data or include only two time points. Data from longitudinal studies, though not as widely available, are preferable because they allow additional questions to be addressed, such as whether the changes in depressive symptoms over time are related to alcohol use. An individual who is experiencing increasing levels of depressive symptoms might be more or less inclined to misuse alcohol relative to one who has experienced stable symptoms over the same time period. Previous studies have explored this question, with inconsistent results: while Marmorstein found that higher initial levels of depressive symptoms were associated with more pronounced increases in alcohol use over time among males, Needham found that, for both males and females, higher depressive symptoms were inversely related to increases in binge drinking over time [3, 12]. Similar to Needham, Clark and colleagues [13] found that, while baseline depressive symptoms were positively associated with later alcohol use, linear growth in depressive symptoms was inversely related to later alcohol use among a large sample of young adolescents.

The current study, based on a large UK cohort, examines the relationship between depressive symptoms reported across adolescence and alcohol phenotypes at age 18 years 8 months. The primary objectives of the current study are to: (1) to derive a longitudinal model of depressive symptoms in early to late adolescence using data spanning 12 years 10 months to 17 years 10 months; and (2) to estimate the relationship between measures characterizing variation and change in depressive symptoms and later alcohol use and harmful use. We hypothesize that higher initial levels of depressive symptoms, and more pronounced growth in these symptoms, will be associated with harmful alcohol use [11, 12]. We further hypothesize that this relationship will be stronger in females, based on previously described subtypes of alcohol dependence [5, 6]. This study will contribute to the extant literature in that it employs a large, generalizable sample that has been assessed consistently for depressive symptoms during a critical developmental time frame for both depression and alcohol use and misuse. Furthermore, the availability of a broad range of other relevant phenotypes, including socio-demographic, familial, and individual risk variables, enables us to control for potential confounding and draw conclusions that are specific to the role of initial levels of and increases in depressive symptoms.

## Method

### Participants

Data were drawn from the Avon Longitudinal Study of Parents and Children (ALSPAC), an ongoing population-based study designed to investigate the effects of a wide range of influences on the health and development of children. Pregnant women residing in the south-west of England, who had an estimated date of delivery between April 1991, and December 1992, were invited to participate. The initial study cohort consisted of 14,062 pregnancies and 13,978 (52 % males and 48 % females) singletons/twins still alive at 12 months of age. Children enrolled in ALSPAC were more educated at 16 compared to the national average and less likely to be eligible for free school meals (an indicator of low income in the UK) [14]. Detailed information about ALSPAC is available via the study website (http://www.bris.ac.uk/alspac) which also contains a fully searchable data dictionary (http://www.bristol.ac.uk/alspac/researchers/data-access/data-dictionary/). Ethical approval for the study was obtained from the ALSPAC Law and Ethics Committee and local Research Ethics Committees. The primary source of data collection was via self-completion questionnaires administered at four points during the prenatal period then at regular intervals following birth to both parents and the “study child”. Since the age of 7 years the whole cohort has been invited to a regular “focus” clinic for a variety of hands-on assessments.

### Measures

#### Depressive symptoms

The Short Mood and Feelings Questionnaire (SMFQ) [15] is a brief (13-item) questionnaire enquiring about the occurrence of depressive symptoms over the past 2 weeks. The SMFQ correlates highly with more extensive depression rating scales like the children’s depression inventory [16] and the diagnostic interview schedule for children [17] and discriminates depressed from non-depressed children in general population samples. Study participants have so far completed the SMFQ on six occasions spanning adolescence, either by filling in a postal questionnaire or by answering the same questions at a PC terminal during a clinic visit. The current analysis will focus on the middle four measures: 12 years 10 months (clinic), 13 years 10 months (clinic), 16 years 6 months (questionnaire) and 17 years 10 months (clinic). Actual age at response varied about each intended time point with an SD of approximately 3 months. The first and sixth SMFQ measures were not utilised for the current analysis as they were collected concurrently either with baseline covariates or the alcohol outcomes. The item ‘I was very restless’ was omitted from the 13-item SMFQ in our models because preliminary models based on the earlier time points suggested that individuals were uncertain about the meaning of this item—response rate for this item was noticeably lower and unlike the other 12 items, endorsement rates did not change monotonically with time.

Responses to the remaining 12 SMFQ items were used to create three continuous scales (parcels) by summing alternate items. This approach was taken to ease the computational burden (compared with using all 48 individual ordinal responses) and to permit the use of maximum likelihood (ML) estimation, with its more realistic missing data assumptions compared to a least-squares estimator. Furthermore, a CFA-based approach using indicators of a latent construct would permit us to consider the impact of non-constant measurement properties of the parcels (see statistical methods for details). We have chosen a dimensional approach to the SMFQ rather than using a cut-point as there is evidence that depression may be better represented as a continuum [18], and that a dimensional approach should be favoured over a categorical approach for hypothesis testing [19].

#### Alcohol outcomes

In a postal questionnaire administered at age 18 years 8 months the young people completed the self-report version of the ten-item alcohol use disorders identification test (AUDIT) [20]. Almost 40 % of individuals in this sample are consuming alcohol at a ‘hazardous’ level (total AUDIT score 8+)—which may be considered as “normative.” We derived two alternative outcome measures. Firstly we used the standard cut point for ‘harmful’ alcohol use (total score 16+) to derive a binary outcome. Respondents answering ‘No’ to the stem question on recent alcohol use were assigned a score of zero and retained in further analyses as members of the non-harmful group. We refer to this measure as “harmful alcohol use”. Secondly, the ten ordinal responses were used to estimate a unidimensional latent variable using confirmatory factor analysis. Whilst these ten items refer to both the use of alcohol and the consequences thereof, for the sake of simplicity we refer to this latent measure as “alcohol use”. We believe that, for this age group in particular, our measure is driven primarily by the initial alcohol-use AUDIT items and this is borne out by correlations in the range 0.76–0.78 between the derived scale and the items of consumption and frequency of use. Through the inclusion of the other AUDIT items we obtain additional information and hence greater precision. Both of these outcome measures are important to public health and policy. Whilst one, to an extent, is a dichotomised version of the other, there are many precedents to this such as continuous BMI versus dichotomous obesity, or blood pressure and hypertension.

#### Other covariates

Additional potentially important variables were selected based on empirical evidence covering a number of different domains, and were adjusted sequentially to examine the incremental impact on the parameter estimates of interest. Unless otherwise indicated, these questions were answered by the young person’s main carer. Socio-demographic variables: data collected by questionnaire during the antenatal period, which comprised housing tenure (coded as owned/mortgaged, privately rented, subsidised housing rented from council/housing-association), maternal educational attainment (coded as no high school qualifications, high school, beyond high school), and parity (coded as whether study child is 1st/2nd/3rd child or greater). Maternal variables consisted of maternal smoking at offspring age 12 years (yes/no), maternal alcohol consumption at offspring age 12 years (evidence of bingeing and high weekly consumption derived from detailed record of beers, wines and spirits consumed in previous week), maternal cannabis use at offspring age 9 years (yes/no) and maternal depressive symptoms as measured by questionnaire when the offspring were age 11. Young person’s variables comprised conduct problems at 11 years (score of 0–1/2–3/4+ on the conduct problems subscale of the maternal-report Strengths and Difficulties Questionnaire [21]; and experience of bullying measured during the 13 years clinic (self-report) [22]. Finally risky behaviours at baseline collected through focus clinic at age 13 years (all self-report), which comprised cigarette use (yes/no), cannabis use (yes/no) and alcohol frequency (none/less than weekly/weekly use).

### Statistical methods

**Fig. 1:**
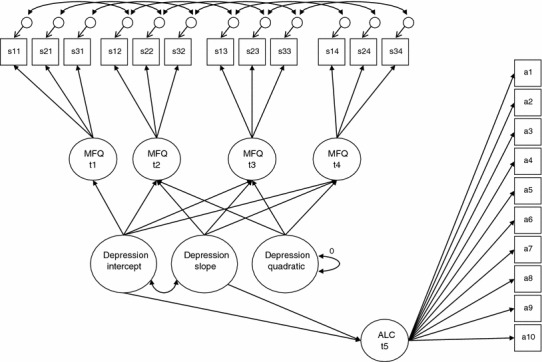
Multiple-indicator growth model with latent measure of alcohol consumption as distal outcome

To model the heterogeneity in the longitudinal patterns of depressive symptoms, a set of multiple-indicator (aka second-order) growth models were applied to these data, with each triplet of continuous scales forming the indicators for an underlying depressive symptoms latent trait at that time point (Fig. 1). Comparisons were made between a linear growth model (IS where I represents the intercept and S represents the slope), a quadratic growth model (ISQ where Q represents the quadratic) and a revised quadratic model with no quadratic variance/covariance terms (ISQ*). Summary fit measures were assessed along with residuals for the mean and covariance structures. Age was parameterized such that the intercept corresponded to symptoms at age 13 (hereafter referred to as baseline). Inclusion of actual age at response had no effect on the estimated growth models so models shown have a fixed age of response at each time point. All models were run separately by sex as the development of depressive symptoms through adolescence has previously been shown to differ between males and females [23, 24]. Formal statistical tests were used, where appropriate, to assess gender differences. Following the estimation of these growth models, the association between the growth factors characterizing the change in depressive symptoms and the later alcohol outcomes was then estimated. Figure 1 illustrates the inclusion of a distal latent trait alcohol outcome. Potential confounders (described above) for this relationship were then introduced. All analyses were carried out using Mplus [25] version 7.0 using the MLR estimator.

#### Measurement invariance

To investigate possible lack of measurement invariance in these data we followed the steps recommended in Topic 4 of Mplus’ online course [26]. Following the estimation of single-indicator growth models utilising each of the three indicators in turn as well as their sum (i.e. the sum of all 12 items), evidence for a lack of measurement invariance was sought through a series of confirmatory factory analysis models in which loadings and/or intercepts were permitted to vary across time. Whilst traditional fit-statistics (CFI/TLI/RMSEA) indicated that the fully invariant model was acceptable, some residuals were high under this degree of constraint. An approach in which loadings and intercepts were constrained/freed consecutively resulted in partially invariant models with improved residuals in comparison to their fully-invariant counterparts (Supplementary Table X1). Whilst these refined models were slightly better fitting to the data, this additional complexity had a negligible impact on the models results hence we focus here on the fully-invariant results, with more complete results available as supplementary material.

#### Missing data

Estimation was carried out using the MLR estimator which incorporates a likelihood-based approach to the problem of missing data [27]. Any respondent with at least one repeated measure may be included under the missing at random (MAR) assumption which states that the probability of a particular variable being missing can depend on other variables in the model, but not the actual unmeasured value of the variable in question. When estimating the growth models, we compared the results obtained from a complete case (CC) analysis with those obtained in samples with one, two or three missing values to assess the robustness of the findings (i.e. the shape of the resulting latent trajectory). As the inclusion of the later alcohol outcome data led to a further reduction in the sample available for analysis, we considered an additional complementary missing data treatment when adding distal outcomes to the growth model. The results obtained when restricting the analyses to those with alcohol data were compared with a second bias-adjusted set derived using inverse probability weighting (IPW) [28]. This method was employed in preference to extending the likelihood-based method to cover missingness in the outcome since it was felt less likely that the MAR assumption would hold for these data. For the bias-adjustment, logistic regression models were estimated to predict inclusion in the final sample using a number of indicators of family adversity in childhood (good predictors of subsequent dropout). The reciprocal of the predicted probabilities from these models were then used as sampling weights (ranging from 1.8 to 18.2 for some individuals) to adjust the regression models of interest.

## Results

### Sample description and data availability

The starting sample comprised 13,978, and of these 7,870 (56.3 %) provided at least one measure of depressive symptoms. (1,688 respondents provided only one SMFQ measure, 1,788 = 2 SMFQ measures, 1,837 = 3 SMFQ measures, 2,557 = complete set of 4 measures). Table 1 shows strong evidence of a relationship between sociodemographic variables and missing data on depressive symptoms. The main focus for the modelling of depressive symptoms will be those with at least half of the required SMFQ data (2,942 male/3,240 female). Among these, the numbers with follow-up information on alcohol at age 18.5 was further reduced (965 male/1,660 female).

**Table 1:** Demographics against SMFQ data availability

	*n*	Data availability					*χ* ^2^, *p*
		No SMFQ data (*n* = 6,112) (%)	1 measure (*n* = 1,682) (%)	2 measures (*n* = 1,788) (%)	3 measures (*n* = 1,837) (%)	4 measures (*n* = 2,557) (%)	
Sex							
Male	7,220	56.8	47.9	56.8	47.4	41.3	*χ* ^2^ = 217.6, *p* < 0.001
Female	6,756	43.2	52.1	43.2	52.6	58.7	
Housing tenure							
Mortgaged/owned	9,559	61.2	73.7	80.0	83.1	88.2	*χ* ^2^ = 868.4, *p* < 0.001
Private rented	1,384	13.7	11.6	8.4	8.2	6.7	
Subsidized rented	2,082	25.1	14.7	11.7	8.7	5.2	
Parity							
First born	5,770	41.7	41.6	43.2	49.0	50.8	*χ* ^2^ = 124.7, *p* < 0.001
Second born	4,539	34.7	36.4	37.1	34.6	34.2	
Third born plus	2,618	23.6	22.0	19.8	16.4	15.0	
Maternal education							
A level or higher	4,392	24.7	32.6	34.1	42.8	53.3	*χ* ^2^ = 900.1, *p* < 0.001
O-level	4,296	34.0	34.2	38.2	36.3	32.4	
<O-level	3,728	41.3	33.3	27.7	20.9	14.3	
Household income							
Top 20 %	1,992	15.1	17.6	18.6	22.7	27.6	*χ* ^2^ = 366.2, *p* < 0.001
Middle 60 %	5,937	56.1	60.0	61.8	62.3	61.4	
Lowest 20 %	2,010	28.8	22.4	19.6	15.1	11.0	
Social class							
Professional/managerial and tech.	6,339	46.6	52.2	55.3	59.2	69.0	*χ* ^2^ = 334.6, *p* < 0.001
Skilled non-manual or lower	5,162	53.4	47.8	44.7	40.8	31.0	
Maternal age at delivery							
<25 years	3,337	33.8	21.9	18.7	14.8	11.6	*χ* ^2^ = 807.5, *p* < 0.001
25–29	5,403	37.4	40.5	42.2	40.4	36.7	
30–34	3,850	21.3	27.6	29.6	33.2	37.0	
35+	1,386	7.5	10.1	9.4	11.7	14.7	

### Simple descriptive analysis of sum scores

A preliminary investigation focussed on single scales created from summing all 12 on SMFQ items (possible total score zero to 24). Here there was strong evidence of a sex difference (*p* < .001) across all four time points with females scoring consistently higher than males. The difference was most marked at the third time point (16 years 6 months). Estimated sex difference at 12 years 10 months = 0.56 points [0.41, 0.72]; difference at 13 years 10 months = 1.11 [0.93, 1.29]; difference at 16 years 6 months = 1.75 [1.50, 1.99], difference at 17 years 10 months = 0.96 [0.71, 1.21]. Estimated mean SMFQ scores were minimally affected when restricting the sample to those with complete symptom data and this sample restriction also had little impact on the sex differences observed.

### Growth models for depressive symptoms

**Fig. 2:**
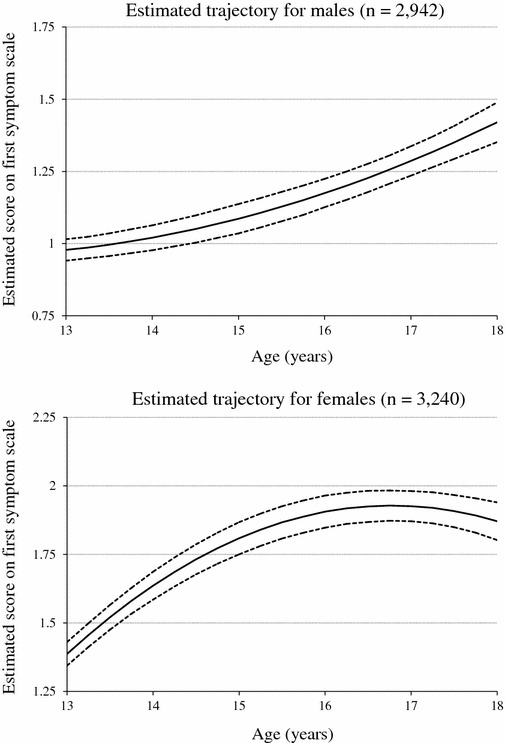
Estimated trajectories of depressive symptoms across adolescence (first MFQ indicator) for (**i**) males and (**ii**) females with data for at least 2 waves

On the basis of summary fit statistics along with residuals for the mean and covariance structures, a quadratic model with zero variance and covariances for the quadratic growth factor was deemed acceptable for both sexes. Males: CFI = 0.973, TLI = 0.964, RMSEA = 0.045, females: CFI = 0.965, TLI = 0.953, RMSEA = 0.060. More details on model fit can be found in Supplementary Table X2. Figure 2 shows estimated trajectory for depressive symptoms complete with its 95 % confidence interval for this model. Since each indicator would be expected to exhibit its own trajectory, we illustrate with the first indicator only. Under assumption of parameter invariance all three indicators display parallel trajectories through time, from different levels at baseline.

For females, change in depressive symptom levels was described by a strong positive slope and a negative quadratic whilst for males change consisted of a more moderate positive slope and a positive quadratic. For both sexes there was strong evidence of variation in symptom levels at baseline [female intercept variance = 0.74 (SE = 0.048), male intercept variance = 0.52 (SE = 0.045)], and also of variation in symptom change, described by the slope variance [female slope variance = 3.22 (SE = 0.373), male slope variance = 2.65 (SE = 0.330)]. Tests of parameter differences between males and females were carried out using a single-sample analysis, stratified by gender. In terms of the growth factor covariance matrix, there was strong evidence (*p* < 0.001) of greater intercept variance for females but little evidence (*p* ~ 0.2) for a difference in either slope variance or intercept/slope covariance. There was strong evidence (*p* < 0.001) for gender differences in all three growth factor means, as is apparent from the trajectory forms in Fig. 2. Results were consistent when varying the amount of missing data permitted.

### Change in depressive symptoms and later alcohol use and harmful use

Information on alcohol use at age 18½ was available for 3,100 participants, and all but 109 of these had provided at least one earlier measure of depressive symptoms. Among the sample of 3,100 there was little evidence of an association between harmful use and gender with 8.8 % of males and 7.4 % of females classified as drinking at a harmful level (*χ* ^2^ = 2.0, *p* = 0.153). The pattern was similar when restricting the sample to those with 2 or more depressive symptom measures (8.1 % of males, 7.1 % of females, *χ* ^2^ = 0.84, *p* = 0.360).

#### Regression models

Table 2 shows the association between baseline and change in depressive symptoms and later alcohol outcomes, for males and females, obtained from the measurement-invariant model. A comparison of results for this model, a partially-invariant model as well as a simpler single-indicator model using 12-item sum-scores, can be found in Supplementary Tables X3 and X4. As these are results from one-step models, the intercept and slope terms are mutually adjusted for.

**Table 2:** Association between intercept and slope for depressive symptoms and later alcohol use and harmful use (multiple-indicator, fully invariant model)

	Unadjusted	Adjusted 1	Adjusted 2	Adjusted 3
	(*N* = m:965/f:1,660)	(*N* = m:927/f:1,577)	(*N* = m:734/f:1,177)	(*N* = m:619/f:1,018)
Continuous trait alcohol use outcome^a^				
Male				
Intercept for depressive symptoms	0.03 [−0.07, 0.13]	0.04 [−0.07, 0.14]	0.05 [−0.07, 0.16]	0.02 [−0.11, 0.16]
	*p* = 0.563	*p* = 0.481	*p* = 0.426	*p* = 0.754
Slope for depressive symptoms	−0.04 [−0.16, 0.08]	−0.02 [−0.14, 0.10]	−0.03 [−0.17, 0.10]	−0.01 [−0.16, 0.14]
	*p* = 0.525	*p* = 0.770	*p* = 0.610	*p* = 0.892
Female				
Intercept for depressive symptoms	0.04 [−0.04, 0.12]	0.06 [−0.02, 0.14]	0.04 [−0.05, 0.13]	0.01 [−0.10, 0.11]
	*p* = 0.299	*p* = 0.169	*p* = 0.432	*p* = 0.895
Slope for depressive symptoms	0.12 [0.02, 0.22]	0.12 [0.02, 0.22]	0.15 [0.04, 0.25]	0.14 [0.03, 0.25]
	*p* = 0.023	*p* = 0.021	*p* = 0.007	*p* = 0.013
Dichotomous harmful alcohol use outcome^b^				
Male				
Intercept for depressive symptoms	1.17 [1.02, 1.35]	1.17 [1.03, 1.34]	1.19 [1.02, 1.39]	1.10 [0.91, 1.33]
	*p* = 0.029	*p* = 0.020	*p* = 0.024	*p* = 0.307
Slope for depressive symptoms	1.01 [0.82, 1.23]	1.05 [0.87, 1.27]	1.04 [0.84, 1.29]	1.07 [0.85, 1.34]
	*p* = 0.954	*p* = 0.623	*p* = 0.728	*p* = 0.586
Female				
Intercept for depressive symptoms	1.29 [1.15, 1.45]	1.31 [1.16, 1.47]	1.29 [1.12, 1.48]	1.39 [1.18, 1.64]
	*p* < 0.001	*p* < 0.001	*p* < 0.001	*p* < 0.001
Slope for depressive symptoms	1.22 [1.05, 1.41]	1.23 [1.07, 1.42]	1.26 [1.07, 1.48]	1.24 [1.06, 1.46]
	*p* = 0.010	*p* = 0.005	*p* = 0.005	*p* = 0.007

Adjusted 1: adjusted for maternal education, parity and tenure

Adjusted 2: further adjusted for maternal data: smoking @12, alcohol @12, cannabis @9, EPDS @11

Adjusted 3: further adjusted for YP data: conduct problems @11, bullying @13, smoking, cannabis and alcohol @13

m:/f: indicate sample sizes for each gender

^a^Estimates are standardized regression coefficients with 95 % CI. Indicate SD change in outcome for 1 SD change in exposure

^b^Estimates are odds ratios with 95 % CI. Refer to change in odds of outcome for 1 SD change in exposure

##### Alcohol use

For the continuous trait alcohol use outcome, estimates indicate the change in SDs per one SD change in either intercept or slope of depressive symptoms. For males, results show little evidence of a relationship between baseline levels or change in depressive symptoms and later alcohol use. In females, there is weak evidence (*p* ~ 0.02) of an association for change in depression symptoms, however this effect is small in magnitude. Adjustment for potential confounders has little effect on these estimates.

##### Harmful alcohol use

For the binary outcome measure of harmful use, estimates indicate the odds-ratio for a one-SD change in depression intercept or slope. For males there is weak evidence (*p* ~ 0.02) of an association between depressive symptoms at baseline and harmful use at 18 years 8 months. This association is weak with only a 17 % increase in odds per one SD change in symptom level. Adjusting for confounders has little effect until the final adjustment in which the already-moderate estimates are attenuated considerably. The primary source of this attenuation was the measure of conduct problems, with baseline depressive symptoms having little effect once this was included in the multivariable model. In contrast to the males’ results, there is evidence of a stronger association in females for both depression intercept and slope. A one SD difference in baseline depression represents almost a 30 % increased odds of harmful drinking, with a slightly more moderate increase of 22 % associated with a one SD difference in symptom slope, i.e. both baseline and change appear to confer a risk of harmful drinking. Estimates appear robust to the adjustment for covariates, with the exception of adjustment for bullying which led the estimates to increase slightly.

#### Missing data

As previously described, an inverse probability weighting approach was employed to adjust for the potential bias in the final models due to the attenuation in sample size following the inclusion of outcome data. A comparison of the results with and without this adjustment can be found in Supplementary Table X5. These figures demonstrate only minor alteration when this differential dropout was accounted for.

## Discussion

We have used data from a large prospective UK birth cohort to derive a longitudinal latent variable model describing depressive symptoms in early to late adolescence, and to estimate the relationship between depressive symptoms growth factors and alcohol use and harmful use at 18 years 8 months.

### Depressive symptom trajectories

Depressive symptoms increased across time, and model fitting revealed that a curvilinear function was adequate to describe these changes, with the shape of the curve differing across the sexes. Among males, depressive symptoms increased gradually at first, followed by a steeper increase in later adolescence, whilst among females, depressive symptoms increased more markedly in early adolescence before stabilizing as adulthood approached. The trajectories of depressive symptoms are largely consistent with previous reports. In particular, the rapid increase in symptoms, followed by a plateau, observed in females is similar to that observed in other population-based samples [3, 29–31]. Findings for males’ trajectories have been more varied, with some studies reporting consistently low levels of depressive symptoms [29, 30], others finding a shape similar to females in the current sample [3], or gradually decreasing symptoms [32, 33]. Consistent with previous studies [23, 24], initial levels of depressive symptoms are similar across the sexes; however, sex-specific mean symptom levels quickly diverge.

### Depressive symptoms and later alcohol use

The relationship between alcohol outcomes and both baseline and change in depressive symptoms differed across sex, with females demonstrating a stronger association which proved more robust to adjustment for covariates. Among males, baseline levels of depressive symptoms were positively associated with later harmful alcohol use; however, change in depressive symptoms across the adolescent period was related to neither use nor harmful use. Observed associations were substantially attenuated in fully adjusted models, particularly when childhood conduct problems were accounted for. Among females, change in depressive symptoms was strongly positively associated with both alcohol use and harmful use. In addition, baseline level of depressive symptoms was positively associated with harmful use. Taken together, these findings provide evidence that the relationship between adolescent depressive symptoms and later alcohol use differs across sex and alcohol outcome. Generally, the associations identified in the current study are consistent with previous reports of positive associations between depressive symptoms and alcohol use [8–11, 34–36], though not all previous work has employed longitudinal data or conducted analyses separately by sex and hence the current results could explain some inconsistencies in some previous work.

### Strengths and limitations

This study has a number of strengths. Firstly, through the use of repeated measures of self-reported depressive symptoms, this study was able to identify of trajectories of these symptoms from early through late adolescence and relate these longitudinal patterns with later alcohol behaviour. This is in contrast to previous studies that have been limited to cross-sectional or a single follow-up [8–11]. Secondly, the availability of data from the AUDIT has permitted us to explore whether the relationship between depressive symptoms and alcohol phenotypes differed depending on the nature of the phenotype (i.e., use vs. harmful use). The presence and strength of the associations between depressive symptoms and alcohol outcomes varied across the sexes; across facets of depressive symptoms (intercept vs. slope); and across alcohol outcomes (use vs. harmful use).

Nevertheless, there are a number of limitations to these analyses which should be considered. First, as with any longitudinal study, missing data becomes more extensive at each successive stage of data collection. Attrition rates frequently differ across socio-demographic variables, and could have had a modest impact on our results. Furthermore, although we did observe differences in demographic measures depending on data availability (as described in Table 1), the results presented were comparable to results from analyses that used only individuals for whom complete data were available. Secondly, the sample used in these analyses is primarily of European ethnicity, all of whom were born within a specified geographic area; results may not generalize to regions with a markedly different ethnic make-up. An additional limitation is the use of only one reporter (in this case self-report) of depressive symptoms and alcohol use, which could hence result in reporter bias. Finally, due to the timing of data collection as well as the longitudinal consistency of the alcohol measures administered, we were unable to account for concurrent alcohol in these models. It is likely, particularly when considering the females’ findings that the increase in depressive symptoms is running in tandem with a parallel increase in alcohol use and misuse. This concurrent association may be complex, with a bi-directional aspect operating on a much finer timescale than might be elucidated using annually collected panel data. Unfortunately, concurrent consistent measures of alcohol use are not available, precluding our ability to address this possibility. We were also unable to conduct cross-lag analyses due to inconsistent alcohol measures across the age range of interest.

### Mechanisms and implications

In females, rising levels of depressive symptoms from early to late adolescence were strongly associated with increases in both normative and harmful alcohol use at 18 years. Females with higher levels of depressive symptoms at age 13 were also more likely to engage in harmful alcohol use. This finding leads to speculation about the potential mechanisms linking depressive symptoms to later alcohol use. Previous work has shown that earlier and steeper increases in depressive symptoms are found in females who experience earlier puberty than their peers [37]. Early maturing females may have increased opportunities to engage in alcohol consumption due to their tendency to socialise with females and males who are older [38]. The tendency for early maturing females to engage in romantic relationships at a younger age than their peers (often before they are emotionally ready) could make them particularly vulnerable to depression [39]. Depressive symptoms during adolescence could lead to young people ‘self-medicating’ as a way of coping with these symptoms and increasing risk for developing problematic alcohol use.

As females’ depressive symptoms escalate, they may be more sensitive to other social stimuli that might promote drinking. Kelly and colleagues [40] found that depression was among the variables that mediated the relationship between peer drinking and early adolescent drinking, though these analyses were not conducted separately by sex. Other evidence suggests that social learning and alcohol expectancies—which are likely influenced by one’s own drinking experiences as well as by his or her peers—might moderate the relationship between depression and alcohol use during adolescence [41], but again, the authors did not explore whether this effect differs by sex. Biological influences underlying females’ depressive symptoms during this period could differ from those underlying males’ symptoms and weak evidence for this have been shown previously [42]; these factors could be related in turn to females’ liability to misuse alcohol.

In contrast to the finding for females, there was no evidence that increasing levels of depressive symptoms in males was related to harmful alcohol use at 18 years. There was evidence for a moderate association between baseline depressive symptoms and harmful alcohol use, but this was substantially attenuated after adjusting for conduct problems. In males the rise in depressive symptoms was seen to occur at a later age and was of a reduced magnitude compared with the rise seen in females. Consequently, our measures of depressive symptoms may be too early or too insensitive for an association in males to manifest. The impact of adjustment for late childhood conduct problems is worthy of note and offers insight into a potential alternative mechanism through which early adolescent depressive symptoms may confer a risk of later harmful alcohol use.

More generally, depressive and anxiety symptoms could lead to alcohol misuse via expectations about alcohol use, interpersonal skill deficits, and coping motives [4], Hussong and colleagues state that whilst internalizing symptoms might be related to a delay in the onset of alcohol use, e.g. due to social withdrawal [43] which could impact one’s access to peers who use alcohol, such symptoms do not necessarily reduce the risk of alcohol use later in adolescence and beyond [4]. Indeed, many studies have demonstrated that adolescents with current or past symptoms of depression are more likely than their peers to engage in a variety of alcohol-related behaviours such as drinking to intoxication, binge drinking and drinking without parental permission [1, 9, 34–36].

### Overall conclusions and further work

The current study uses longitudinal data from a large, population-based sample, and benefits from the use of standardized instruments for the assessment of depressive symptoms and alcohol use. The latent variable methods applied enable us to examine the association between the effects of mean levels of depressive symptoms, and growth in depressive symptoms, on both the use and harmful use of alcohol. Our findings indicate that these relationships differ across males and females and the alcohol outcome of interest. Despite this variation, increased depressive symptoms during adolescence are generally associated with more alcohol use at 18 years 8 months. The predictive nature of depressive symptoms in both sexes could be helpful for public health and clinical efforts (e.g., education, identification, intervention), as demonstrated by previous findings that a cognitive behavioural intervention approach among teens with depressive symptoms can reduce later substance use [44]. The finding that the nature of the alcohol outcome may be critical in determining whether an association with depressive symptoms exists is an important one, and should be considered when comparing the results of studies that examine the relationships between depressive symptoms and alcohol use. The psychological and/or biological mechanisms underlying these associations should be the focus of additional research.

## Electronic supplementary material

Below is the link to the electronic supplementary material.

Supplementary material 1 (DOCX 38 kb)
