# Supplementary material for: The association between depressive symptoms from early to late adolescence and later use and harmful use of alcohol
Source: Eur Child Adolesc Psychiatry. 2014 Aug 18;23(12):1219–30. doi: 10.1007/s00787-014-0600-5 (PMC4246124; doi:10.1007/s00787-014-0600-5)
Supplement: Supplementary file 1 — Supplementary material 1 (DOCX 38 kb) [file 787_2014_600_MOESM1_ESM.docx]

Table X1. Comparison of CFA models

|  | Male (n = 2,942) | | | | Female (n = 3,240) | | | |
| --- | --- | --- | --- | --- | --- | --- | --- | --- |
|  | (i) | (ii) | (iii) | (iv) | (i) | (ii) | (iii) | (iv) |
| # Parameters | 60 | 54 | 48 | 53 | 60 | 54 | 48 | 57 |
| CFI | 1.000 | 1.000 | 0.987 | 1.000 | 1.000 | 0.997 | 0.977 | 1.000 |
| TLI | 1.000 | 0.999 | 0.979 | 1.000 | 1.000 | 0.995 | 0.964 | 1.000 |
| ssa-BIC | 73976.6 | 73960.4 | 74116.9 | 73947.8 | 93472.5 | 93500.8 | 93859.3 | 93458.7 |
| RMSEA | 0.000 | 0.005 | 0.034 | 0.000 | 0.002 | 0.019 | 0.053 | 0.000 |
|  |  |  |  |  |  |  |  |  |
| *Mean structure* |  |  |  |  |  |  |  |  |
| Lowest residual | -0.002 | -0.003 | -0.08 | -0.011 | -0.001 | -0.001 | -0.122 | -0.005 |
| Highest residual | 0.001 | 0.001 | 0.151 | 0.014 | 0.001 | 0.002 | 0.2 | 0.007 |
| # normalised residuals > 1.96 | 0 | 0 | 3 | 0 | 0 | 0 | 5 | 0 |
|  |  |  |  |  |  |  |  |  |
| *Covariance structure* | |  |  |  |  |  |  |  |
| Lowest residual | -0.058 | -0.066 | -0.060 | -0.069 | -0.06 | -0.107 | -0.139 | -0.060 |
| Highest residual | 0.074 | 0.073 | 0.056 | 0.076 | 0.056 | 0.118 | 0.145 | 0.055 |
| # normalised residuals > 1.96 | 0 | 0 | 0 | 0 | 0 | 0 | 1 | 0 |

1. Fully variant model:

all intercepts freely estimated and all loadings freely estimated for scales 2 and 3

1. Invariant loadings model:

all intercepts freely estimated and all loadings constrained to be equal across time for each scale.

1. Fully invariant model:

all intercepts / loadings constrained to be equal across time for each scale. Factor means estimated for t2, t3, t4.

1. Partially invariant model:

To obtain the partially invariant models the first step was a fully invariant model with all loadings and intercepts freely estimated apart from the loadings for the indicator-1. Loadings were examined first, with equality constraints applied successively until there was statistical evidence of differences for all remaining unconstrained loadings. Intercept invariance was assessed subsequently. The scale was *anchored* together by fixing the intercepts for the first indicator. Successive models were then examined in which pairs of intercepts were constrained to be equal. Constraints were only considered for pairs of scales where loadings were found to be invariant.

Table X2. Model fit for multiple-item growth models

|  | Male (n = 2,942) | | | | | Female (n = 3,240) | | | | |
| --- | --- | --- | --- | --- | --- | --- | --- | --- | --- | --- |
|  | I | IS | ISQ* | ISQ | ISQ* (pv) | I | IS | ISQ* | ISQ | ISQ* (pv) |
| # Parameters | 37 | 40 | 41 | 44 | 53 | 37 | 40 | 41 | 44 | 57 |
| CFI | 0.935 | 0.972 | 0.973 | 0.975 | 1.000 | 0.919 | 0.961 | 0.965 | 0.969 | 1.000 |
| TLI | 0.919 | 0.964 | 0.964 | 0.963 | 1.000 | 0.899 | 0.948 | 0.953 | 0.956 | 1.000 |
| Sample size adjusted BIC | 74852.3 | 74309.8 | 74306.8 | 74285.1 | 73947.8 | 94999.7 | 94174.9 | 94098.8 | 94014.8 | 93458.7 |
| RMSEA | 0.067 | 0.045 | 0.045 | 0.045 | 0.000 | 0.088 | 0.063 | 0.060 | 0.058 | 0.000 |
|  |  |  |  |  |  |  |  |  |  |  |
| *Mean structure* |  |  |  |  |  |  |  |  |  |  |
| Lowest residual | -0.203 | -0.131 | -0.114 | -0.113 | -0.011 | -0.403 | -0.223 | -0.124 | -0.122 | -0.005 |
| Highest residual | 0.466 | 0.214 | 0.184 | 0.187 | 0.014 | 0.452 | 0.166 | 0.194 | 0.199 | 0.005 |
| # normalised residuals > 1.96 | 10/12 | 5/12 | 5/12 | 5/12 | 0/12 | 10/12 | 8/12 | 5/12 | 5/12 | 0/12 |
|  |  |  |  |  |  |  |  |  |  |  |
| *Covariance structure* |  |  |  |  |  |  |  |  |  |  |
| Lowest residual | -0.312 | -0.174 | -0.173 | -0.146 | -0.146 | -0.546 | -0.406 | -0.406 | -0.227 | -0.06 |
| Highest residual | 0.379 | 0.145 | 0.147 | 0.138 | 0.138 | 0.617 | 0.446 | 0.453 | 0.326 | 0.055 |
| # normalised residuals > 1.96 | 34/12 | 12/78 | 11/78 | 7/78 | 0/78 | 42/78 | 36/78 | 33/78 | 22/78 | 0/78 |

I : Random intercept model with no growth

IS: Random intercept/slope

ISQ: Random intercept/slope/quadratic

ISQ*: Random intercept/slope/quadratic but with fixed effect only for quadratic term (no quadratic variance/covariances)

ISQ*(pv): As above but with partial invariance of some loadings/intercepts

Table X3. Model comparison for the association between intercept and slope for depressive symptoms and later alcohol use and harmful use (Male).

|  |  | Unadjusted | Adjusted 1 | Adjusted 2 | Adjusted 3 |
| --- | --- | --- | --- | --- | --- |
|  |  | (N=965) | (N=927) | (N=734) | (N=619) |
| **Continuous trait alcohol use outcome †** | |  |  |  |  |
| *Single-indicator growth model* | Intercept for depressive symptoms | 0.04 [-0.06, 0.13] | 0.04 [-0.06, 0.14] | 0.04 [-0.07, 0.15] | 0.03 [-0.10, 0.15] |
|  |  | p = 0.471 | p = 0.454 | p = 0.451 | p = 0.676 |
|  | Slope for depressive symptoms | -0.03 [-0.15, 0.08] | -0.01 [-0.13, 0.11] | -0.02 [-0.15, 0.11] | 0.00 [-0.14, 0.14] |
|  |  | p = 0.600 | p = 0.876 | p = 0.740 | p = 0.998 |
|  |  |  |  |  |  |
| *Multiple-indicator fully invariant growth model* | Intercept for depressive symptoms | 0.03 [-0.07, 0.13] | 0.04 [-0.07, 0.14] | 0.05 [-0.07, 0.16] | 0.02 [-0.11, 0.16] |
|  |  | p = 0.563 | p = 0.481 | p = 0.426 | p = 0.754 |
|  | Slope for depressive symptoms | -0.04 [-0.16, 0.08] | -0.02 [-0.14, 0.10] | -0.03 [-0.17, 0.10] | -0.01 [-0.16, 0.14] |
|  |  | p = 0.525 | p = 0.770 | p = 0.610 | p = 0.892 |
|  |  |  |  |  |  |
| *Multiple-indicator partially-invariant growth model* | Intercept for depressive symptoms | 0.03 [-0.07, 0.13] | 0.04 [-0.07, 0.14] | 0.05 [-0.07, 0.16] | 0.02 [-0.11, 0.16] |
|  |  | p = 0.591 | p = 0.488 | p = 0.438 | p = 0.760 |
|  | Slope for depressive symptoms | -0.04 [-0.15, 0.08] | -0.02 [-0.14, 0.10] | -0.03 [-0.16, 0.10] | -0.01 [-0.16, 0.14] |
|  |  | p = 0.556 | p = 0.761 | p = 0.614 | p = 0.908 |
|  |  |  |  |  |  |
| **Dichotomous harmful alcohol use outcome ‡** | |  |  |  |  |
| *Single-indicator growth model* | Intercept for depressive symptoms | 1.17 [1.02, 1.34] | 1.17 [1.03, 1.33] | 1.19 [1.02, 1.38] | 1.10 [0.92, 1.32] |
|  |  | p = 0.021 | p = 0.017 | p = 0.027 | p = 0.279 |
|  | Slope for depressive symptoms | 1.02 [0.84, 1.23] | 1.06 [0.88, 1.28] | 1.05 [0.85, 1.30] | 1.08 [0.86, 1.35] |
|  |  | p = 0.861 | p = 0.512 | p = 0.666 | p = 0.509 |
|  |  |  |  |  |  |
| *Multiple-indicator fully invariant growth model* | Intercept for depressive symptoms | 1.17 [1.02, 1.35] | 1.17 [1.03, 1.34] | 1.19 [1.02, 1.39] | 1.10 [0.91, 1.33] |
|  |  | p = 0.029 | p = 0.020 | p = 0.024 | p = 0.307 |
|  | Slope for depressive symptoms | 1.01 [0.82, 1.23] | 1.05 [0.87, 1.27] | 1.04 [0.84, 1.29] | 1.07 [0.85, 1.34] |
|  |  | p = 0.954 | p = 0.623 | p = 0.728 | p = 0.586 |
|  |  |  |  |  |  |
| *Multiple-indicator partially-invariant growth model* | Intercept for depressive symptoms | 1.17 [1.02, 1.35] | 1.18 [1.03, 1.34] | 1.20 [1.03, 1.40] | 1.11 [0.92, 1.34] |
|  |  | p = 0.024 | p = 0.017 | p = 0.019 | p = 0.286 |
|  | Slope for depressive symptoms | 1.01 [0.83, 1.23] | 1.05 [0.87, 1.27] | 1.04 [0.84, 1.28] | 1.07 [0.85, 1.35] |
|  |  | p = 0.934 | p = 0.599 | p = 0.731 | p = 0.571 |

Adjusted 1: adjusted for maternal education, parity and tenure.

Adjusted 2: further adjusted for maternal data: smoking @12, alcohol @12, cannabis @9, EPDS@11.

Adjusted 3: further adjusted for YP data: conduct problems @11, bullying@13, smoking, cannabis and alcohol@13.

†: Estimates are standardized regression coefficients with 95% CI. Indicate SD change in outcome for 1 SD change in exposure

‡: Estimates are Odds Ratios with 95% CI. Refer to change in odds of outcome for 1 SD change in exposure

Table X4. Model comparison for the association between intercept and slope for depressive symptoms and later alcohol use and harmful use (Female).

|  |  | Unadjusted | Adjusted 1 | Adjusted 2 | Adjusted 3 |
| --- | --- | --- | --- | --- | --- |
|  |  | N=(1660) | N=(1577) | N=(1177) | N=(1018) |
| **Continuous trait alcohol use outcome †** | |  |  |  |  |
| *Single-indicator growth model* | Intercept for depressive symptoms | 0.04 [-0.03, 0.12] | 0.06 [-0.02, 0.13] | 0.04 [-0.04, 0.12] | 0.01 [-0.08, 0.11] |
|  |  | p = 0.249 | p = 0.146 | p = 0.354 | p = 0.773 |
|  | Slope for depressive symptoms | 0.14 [0.04, 0.23] | 0.14 [0.04, 0.23] | 0.15 [0.05, 0.26] | 0.15 [0.04, 0.26] |
|  |  | p = 0.009 | p = 0.008 | p = 0.005 | p = 0.006 |
|  |  |  |  |  |  |
| *Multiple-indicator fully invariant growth model* | Intercept for depressive symptoms | 0.04 [-0.04, 0.12] | 0.06 [-0.02, 0.14] | 0.04 [-0.05, 0.13] | 0.01 [-0.10, 0.11] |
|  |  | p = 0.299 | p = 0.169 | p = 0.432 | p = 0.895 |
|  | Slope for depressive symptoms | 0.12 [0.02, 0.22] | 0.12 [0.02, 0.22] | 0.15 [0.04, 0.25] | 0.14 [0.03, 0.25] |
|  |  | p = 0.023 | p = 0.021 | p = 0.007 | p = 0.013 |
|  |  |  |  |  |  |
| *Multiple-indicator partially-invariant growth model* | Intercept for depressive symptoms | 0.05 [-0.02, 0.13] | 0.07 [-0.01, 0.14] | 0.05 [-0.04, 0.13] | 0.01 [-0.09, 0.11] |
|  |  | p = 0.152 | p = 0.086 | p = 0.274 | p = 0.796 |
|  | Slope for depressive symptoms | 0.11 [0.01, 0.21] | 0.12 [0.02, 0.21] | 0.14 [0.04, 0.25] | 0.15 [0.04, 0.26] |
|  |  | p = 0.027 | p = 0.023 | p = 0.008 | p = 0.006 |
|  |  |  |  |  |  |
| **Dichotomous harmful alcohol use outcome ‡** | |  |  |  |  |
| *Single-indicator growth model* | Intercept for depressive symptoms | 1.29 [1.15, 1.43] | 1.31 [1.17, 1.46] | 1.29 [1.13, 1.47] | 1.38 [1.19, 1.62] |
|  |  | p < 0.001 | p < 0.001 | p < 0.001 | p < 0.001 |
|  | Slope for depressive symptoms | 1.22 [1.06, 1.41] | 1.24 [1.08, 1.42] | 1.26 [1.08, 1.48] | 1.25 [1.07, 1.46] |
|  |  | p = 0.007 | p = 0.003 | p = 0.003 | p = 0.005 |
|  |  |  |  |  |  |
| *Multiple-indicator fully invariant growth model* | Intercept for depressive symptoms | 1.29 [1.15, 1.45] | 1.31 [1.16, 1.47] | 1.29 [1.12, 1.48] | 1.39 [1.18, 1.64] |
|  |  | p < 0.001 | p < 0.001 | p < 0.001 | p < 0.001 |
|  | Slope for depressive symptoms | 1.22 [1.05, 1.41] | 1.23 [1.07, 1.42] | 1.26 [1.07, 1.48] | 1.24 [1.06, 1.46] |
|  |  | p = 0.010 | p = 0.005 | p = 0.005 | p = 0.007 |
|  |  |  |  |  |  |
| *Multiple-indicator partially-invariant growth model* | Intercept for depressive symptoms | 1.31 [1.18, 1.46] | 1.33 [1.19, 1.48] | 1.31 [1.15, 1.50] | 1.41 [1.21, 1.65] |
|  |  | p < 0.001 | p < 0.001 | p < 0.001 | p < 0.001 |
|  | Slope for depressive symptoms | 1.23 [1.06, 1.41] | 1.24 [1.08, 1.42] | 1.26 [1.08, 1.48] | 1.25 [1.07, 1.46] |
|  |  | p = 0.006 | p = 0.003 | p = 0.003 | p = 0.005 |

Adjusted 1: adjusted for maternal education, parity and tenure.

Adjusted 2: further adjusted for maternal data: smoking @12, alcohol @12, cannabis @9, EPDS@11.

Adjusted 3: further adjusted for YP data: conduct problems @11, bullying@13, smoking, cannabis and alcohol@13.

†: Estimates are standardized regression coefficients with 95% CI. Indicate SD change in outcome for 1 SD change in exposure

‡: Estimates are Odds Ratios with 95% CI. Refer to change in odds of outcome for 1 SD change in exposure

Table X5. Results following inverse probability weighting. Multiple-indicator parameter-invariant growth models with/without the inclusion of sampling weights for non-response

**Results for males**

|  | Unadjusted (N=965) | | Adjusted 3 (N = 619) | |
| --- | --- | --- | --- | --- |
|  | Un-weighted | Weighted | Un-weighted | Weighted |
| Continuous trait alcohol use outcome † | | | | |
|  |  |  |  |  |
| Intercept | 0.03 [-0.07, 0.13] | 0.05 [-0.06, 0.16] | 0.02 [-0.11, 0.16] | 0.05 [-0.11, 0.21] |
|  | p = 0.563 | p = 0.383 | p = 0.754 | p = 0.513 |
| Slope | -0.04 [-0.16, 0.08] | -0.05 [-0.17, 0.07] | -0.01 [-0.16, 0.14] | -0.03 [-0.18, 0.12] |
|  | p = 0.525 | p = 0.415 | p = 0.892 | p = 0.663 |
|  |  |  |  |  |
| Dichotomous harmful alcohol use outcome ‡ | | | | |
|  |  |  |  |  |
| Intercept | 1.17 [1.02, 1.35] | 1.19 [1.02, 1.38] | 1.10 [0.91, 1.33] | 1.13 [0.92, 1.38] |
|  | p = 0.029 | P = 0.027 | p = 0.307 | P = 0.246 |
| Slope | 1.01 [0.82, 1.23] | 1.01 [0.83, 1.23] | 1.07 [0.85, 1.34] | 1.06 [0.85, 1.33] |
|  | p = 0.954 | P = 0.875 | p = 0.586 | P = 0.596 |

**Results for females**

|  | Unadjusted (N=1660) | | Adjusted 3 (N=1018) | |
| --- | --- | --- | --- | --- |
|  | Un-weighted | Weighted | Un-weighted | Weighted |
| Continuous trait alcohol use outcome † | | | | |
|  |  |  |  |  |
| Intercept | 0.04 [-0.04, 0.12] | 0.04 [-0.04, 0.12] | 0.01 [-0.10, 0.11] | -0.01 [-0.11, 0.10] |
|  | p = 0.299 | p = 0.387 | p = 0.895 | p = 0.863 |
| Slope | 0.12 [0.02, 0.22] | 0.12 [0.01, 0.23] | 0.14 [0.03, 0.25] | 0.14 [0.02, 0.26] |
|  | p = 0.023 | p = 0.030 | p = 0.013 | p = 0.021 |
|  |  |  |  |  |
| Dichotomous harmful alcohol use outcome ‡ | | | | |
|  |  |  |  |  |
| Intercept | 1.29 [1.15, 1.45] | 1.28 [1.13, 1.44] | 1.39 [1.18, 1.64] | 1.36 [1.15, 1.60] |
|  | p < 0.001 | p < 0.001 | p < 0.001 | p < 0.001 |
| Slope | 1.22 [1.05, 1.41] | 1.24 [1.07, 1.44] | 1.24 [1.06, 1.46] | 1.29 [1.11, 1.50] |
|  | p = 0.010 | p = 0.005 | p = 0.007 | p = 0.001 |

Adjusted 1: adjusted for maternal education, parity and tenure; maternal data: smoking @12, alcohol @12, cannabis @9, EPDS@11; and YP data: conduct problems @11, bullying@13, smoking, cannabis and alcohol@13.

†: Estimates are standardized regression coefficients with 95% CI. Indicate SD change in outcome for 1 SD change in exposure

‡: Estimates are Odds Ratios with 95% CI. Refer to change in odds of outcome for 1 SD change in exposure
